# Genome Sequence of Enterovirus D68 from St. Louis, Missouri, USA, 2016

**DOI:** 10.1128/genomeA.01630-16

**Published:** 2017-03-02

**Authors:** Kristine M. Wylie, Todd N. Wylie, Gregory A. Storch

**Affiliations:** a Department of Pediatrics, Washington University School of Medicine, St. Louis, Missouri, USA; b McDonnell Genome Institute, Washington University School of Medicine, St. Louis, Missouri, USA

## Abstract

Enterovirus D68 (EV-D68) was rarely observed prior to a widespread outbreak in 2014. We observed its reemergence in St. Louis in 2016 and sequenced the EV-D68 genomes from two patient samples. The 2016 viruses in St. Louis differed from those we had sequenced from the 2014 outbreak but were similar to other viruses circulating nationally in 2016.

## GENOME ANNOUNCEMENT

Enterovirus D68 (EV-D68) is a member of the family *Picornaviridae*, which are positive sense RNA viruses. This virus was first described in 1962 but remained rare until 2014 when a widespread, global outbreak occurred (1). In 2014, we sequenced strains circulating in St. Louis (2) and developed a rapid assay that could be used for EV-D68 surveillance (3). Using this assay to test a subset of enterovirus/rhinovirus-positive respiratory samples from the Clinical Virology Laboratory at St. Louis Children’s Hospital, we found that EV-D68 reemerged in 2016. This is consistent with surveillance carried out by the Centers for Disease Control and Prevention, which did not detect EV-D68 in the 700 samples they tested in 2015 but detected it sporadically in 2016 (http://www.cdc.gov/non-polio-enterovirus/about/EV-D68.html). We selected two samples for genome sequence analysis.

Total nucleic acid was extracted using the NucliSENS easyMAG automated system (BioMérieux, Marcy l’Etoile, France) and treated with DNase. RNA was reverse transcribed, the second strand was synthesized, and resulting cDNA was amplified as previously described (4, 5). Dual-indexed sequencing libraries were constructed using the Accel NGS 2S kit according to the manufacturer’s protocol (Swift Biosciences, Ann Arbor, MI). The two sequencing libraries were pooled, and viral nucleic acid was enriched using targeted sequence capture with ViroCap (6). Enriched nucleic acids were sequenced on the Illumina MiSeq instrument, generating 2 × 150 bp paired-end sequences (Illumina, Inc., San Diego, CA). Primer and Illumina adapter sequences were trimmed with Flexbar (7). Sequences were assembled using IDBA-UD (8), individual sequences were aligned back to the assembly using BWA mem (9), and contigs and aligned sequence reads were visualized using Tablet (10). Contig sequences were manually reviewed and improved. Genomes were annotated with VIGOR (11, 12).

The two St. Louis viruses showed greater than 99% nucleic acid and predicted amino acid sequence identity to each other and to other 2016 strains that had been deposited into GenBank as of 20 October 2016. The St. Louis viruses, which were from patients with respiratory symptoms, had greater than 99% sequence identity to viruses from patients with acute flaccid paralysis submitted to GenBank in August 2016 by the Centers for Disease Control and Prevention (accession numbers KX675261 and KX625262). The 2016 St. Louis viruses shared only 96% nucleotide sequence identity with the 2014 St. Louis virus, and phylogenetic analysis indicated that the 2016 virus in St. Louis did not derive from the 2014 viruses.

When we sequenced the St. Louis virus in 2014, only seven whole-genome sequences and five additional complete coding sequences were available in GenBank. On 20 October 2016, there were 285 whole-genome or complete coding sequences available. The deposition of genome sequences and metadata including geographic location and associated clinical features will help to increase our understanding of EV-D68 and its patholepidemiology.

### Accession number(s).

This project has been deposited in GenBank under the accession numbers KY292525 and KY292526. The versions described in this paper are the first versions.
